# Dynamic Human Resource Management Improves Arterial Blood Gas Sample Collection Outcomes

**DOI:** 10.1155/jonm/3978908

**Published:** 2026-03-09

**Authors:** Qiaoyu Xu, Yao Zhou, Yanjun Xiang, Yanmin Zhao, Shuzhen Zhao

**Affiliations:** ^1^ Department of Outpatient, West China Hospital, Sichuan University, Chengdu, 610041, China, scu.edu.cn; ^2^ West China School of Nursing, Sichuan University, Chengdu, 610041, China, scu.edu.cn

**Keywords:** arterial blood gas analysis, dynamic human resource management, efficiency, nurse satisfaction, specimen collection

## Abstract

**Aims:**

To explore the effects of dynamic human resource management (DHRM) in improving the quality and efficiency of arterial blood gas (ABG) sample collection and its impact on nurse job satisfaction.

**Background:**

ABG analysis is critical for diagnosing and monitoring critically ill patients, but its accuracy heavily depends on specimen collection quality. Given the dependence of ABG accuracy on collection quality, workload pressures and staffing shortages often undermine performance. DHRM, a flexible staffing model, may optimize resource allocation and enhance outcomes. However, its impact on ABG procedures remains underexplored.

**Methods:**

A quasiexperimental study assessed the DHRM framework’s impact on ABG specimen collection. Using a pre–post design, 1800 specimens from December 2022 (control) were compared with 1800 from March 2023 (observation). Twenty nurses trained in ABG protocols participated. DHRM included dynamic staffing, a dispatcher system, fixed‐zone assignments, and standardized ABG procedures. Outcomes measured operational efficiency (waiting/collection times and delayed testing), specimen quality/safety (first‐attempt success, compliance, and hematoma incidence), and nurse job satisfaction (5‐point Likert scale). Data were analyzed using *t*‐tests, Mann–Whitney *U* tests, and chi‐square tests (*p* < 0.05).

**Results:**

DHRM implementation yielded substantial improvements: patient waiting time reduced by 35% (*p* < 0.001) and collection time by 21% (*p* < 0.001), with no difference in delayed testing (*p* = 0.334). Specimen compliance improved significantly (*p* < 0.05) for the absence of clotting, air bubbles, and adequate blood volume, with hematoma incidence decreasing by 37% (*p* = 0.033). Nurse job satisfaction increased overall by 11.3% (*p* = 0.010), with notable gains (*p* < 0.05) in hospital management, workload, salary and benefits, and personal development.

**Conclusion:**

DHRM significantly enhanced ABG sample collection efficiency, quality, and nurse satisfaction, addressing key managerial challenges in high‐demand clinical settings. Further studies are needed to confirm and extend these benefits across diverse settings.

**Implications for Nursing Management:**

Hospitals could consider adopting DHRM to effectively address staffing challenges, standardize procedures, and strategically link performance to incentives in ABG sampling, thereby fostering improved clinical outcomes and enhanced staff morale.

## 1. Introduction

Arterial blood gas (ABG) analysis is a critical diagnostic tool in clinical settings, offering essential insights into respiratory and metabolic homeostasis, particularly for critically ill patients [1, 2]. This analysis facilitates real‐time evaluation of oxygenation, ventilation, and acid–base balance, which is vital for assessing disease progression, guiding mechanical ventilation strategies, and optimizing therapeutic interventions in intensive care environments [3]. However, the clinical utility of ABG results is highly dependent on preanalytical quality. Inconsistencies in specimen collection and handling, such as air contamination, delayed analysis, or temperature excursions, can induce clinically significant deviations in key parameters including pO_2_, lactate, and acid–base balance, ultimately compromising diagnostic accuracy and therapeutic decisions [4–7].

Tertiary hospitals now face a marked increase in ABG sampling demands, driven by heightened admissions of severe respiratory cases requiring intensive monitoring [3]. This surge in healthcare workload critically strains clinical workflows. In high‐demand settings such as intensive care units (ICUs), nurses frequently manage urgent ABG requests, leading to prolonged turnaround times and compromised specimen quality [8, 9]. These failures reflect deeper leadership and staffing‐governance shortcomings: rigid staffing models and fixed point‐of‐care testing resources repeatedly prove inadequate for fluctuating clinical demands [10–12]. During peak periods, such as morning ICU rounds, leadership often lacks the real‐time visibility and flexible deployment mechanisms needed to prevent resource bottlenecks, further delaying critical diagnostic results and exposing systemic gaps in workforce governance [13].

Traditional approaches to ABG sample collection have prioritized technical skill development and equipment upgrades, often overlooking the systemic impact of adaptive workforce management on operational efficiency. Conventional staffing strategies, while initially responsive to demand fluctuations, reveal inherent limitations under sustained pressure [14, 15]. For instance, widespread adoption of tiered staffing models, where nonspecialized nurses are supervised by critical care experts, has inadvertently introduced risks of procedural inconsistencies and workforce attrition, particularly in high‐acuity environments [16]. Similarly, short‐term solutions, such as temporary staff augmentation or internal redeployment, often fail to stabilize workflows during peak demand periods, exacerbating inefficiencies in specimen collection and processing [17, 18]. These challenges highlight a critical gap in healthcare resource management: the inability to dynamically align human resources with real‐time clinical needs [19].

Dynamic human resource management (DHRM) is a strategic staffing framework that dynamically aligns human resources with real‐time clinical needs. Theoretically, DHRM draws on the resource‐based view (RBV) of the firm, which positions skilled human capital as a rare, valuable, and difficult‐to‐imitate resource that drives sustained organizational advantage and improved clinical performance [20]. It also aligns with systems theory by treating healthcare organizations as interconnected wholes, where systemic influences on workforce behavior must be addressed to enable adaptive, error‐reducing interventions [21]. Most directly, DHRM operationalizes complex adaptive system (CAS) principles: nurses act as autonomous agents guided by simple rules and attractors, producing nonlinear interactions that generate emergent outcomes such as enhanced efficiency, workplace learning, staff satisfaction, and patient safety [22]. Together, these frameworks link flexible, real‐time staffing to both workforce performance and clinical outcomes [23].

DHRM represents a potential advancement over traditional models, directly addressing the limitations of conventional float pools and resource teams [24–26]. Traditional float pools often result in nurse dissatisfaction and fragmented care due to their reactive and generalized staffing practices [27, 28]. DHRM resolves these issues by integrating real‐time clinical demand analytics, fostering role adaptability, and enabling proactive resource allocation [29–32]. Empirical evidence from similar dynamic or algorithm‐supported staffing models already demonstrates reduced response times in emergency departments [33], lower burnout and improved retention during COVID‐19 redeployment surges [17, 34], enhanced nurse–patient assignment efficiency [25, 35], and improved ICU efficiency when nurses with cross‐functional competencies dynamically adjust roles according to urgency [36, 37]. This flexibility effectively increases efficiency during high‐demand scenarios, mitigates risks of skill mismatches inherent in static models, and alleviates burnout caused by uneven workloads. By aligning staffing agility with clinical priorities, DHRM offers a solution that harmonizes the strengths of resource teams with the adaptability essential for modern healthcare environments.

Although extensive research has examined flexible staffing models in acute and critical care, most studies focus on broad workforce optimization, rarely addressing the specific managerial challenges posed by high‐frequency, time‐sensitive, and error‐prone procedures such as ABG sampling. In nursing administration, delays and poor specimen quality in ABG collection represent preventable failures that traditional fixed‐shift and float‐pool models have consistently failed to resolve. This quasiexperimental study aimed to bridge this gap by developing and evaluating a tailored DHRM protocol to enhance operational efficiency, specimen quality and safety, and nurse satisfaction in ABG management within ICUs.

## 2. Methods

### 2.1. Study Design and Setting

This quasiexperimental study, employing a pre–post control group design, was conducted at West China Hospital, a large tertiary institution encompassing 61 departments. Ethical approval was obtained from the Clinical Trial and Biomedical Ethics Committee of West China Hospital of Sichuan University (reference no. 1116), and the study adhered to the Declaration of Helsinki. The primary aim was to evaluate the efficacy of DHRM in optimizing ABG specimen collection workflows. This was achieved by comparing retrospective data from December 2022 (preintervention, referred to as the control group) with prospective data collected in March 2023 (post‐ DHRM implementation, referred to as the observation group).

Although DHRM implementation commenced on January 1, 2023, postintervention data collection was intentionally delayed by 2 months to allow for a familiarization period, thereby mitigating potential bias from initial workflow adjustments and unfamiliarity. During this period, nurse adaptation and learning curves were actively monitored through (1) weekly dispatcher‐nurse feedback meetings that recorded and resolved operational difficulties, (2) daily Electronic Health Record (HER) logs tracking task acceptance rates, response times, and protocol adherence, and (3) monthly audits of specimen compliance and hematoma incidence. These measures confirmed steady improvement and stabilization of all indicators by the third week of February 2023, ensuring that March data reflected mature rather than transitional performance.

A parallel cohort of 20 nurses of the Blood Collection Center’s workforce, participated throughout the study to assess workflow adaptations under DHRM. This cohort included five fixed‐shift nurses dedicated to routine ABG sampling across three daily shifts (morning: 08:00–15:00; afternoon: 15:00–23:00; and night: 23:00–08:00). The remaining fifteen flexible‐duty nurses were outpatient phlebotomists, who were redeployed during inpatient demand surges. All participating nurses were female, aged 22–26 years, and received training in ABG protocols.

### 2.2. Study Population and Specimen Sample

The sample size for this study was determined based on the center’s daily throughput and aimed to ensure adequate statistical power (*β* = 0.8 and *α* = 0.05) to detect clinically meaningful differences while mitigating Type II error risk. To meet this calculated requirement, a total of 3600 ABG specimens were needed. A total of 3600 ABG specimens were analyzed, comprising 1800 specimens collected in the control group and 1800 specimens collected in the observation group.

Patient inclusion criteria for specimen collection comprised hospitalized adults (≥ 18 years) clinically requiring ABG analysis. Informed consent was obtained though it was waived for anonymized retrospective data per institutional review board approval. Exclusion criteria for specimen collection included patients who did not give consent; those with contraindications for arterial blood sampling, such as impalpable pulse or negative Allen’s test in the upper extremities, infection or fistula at the desired puncture site, or severe coagulation disorders; samples with an interval exceeding 10 min between arterial and venous sampling; inappropriate sample transfer to the laboratory; and samples from postcardiac arrest patients [38].

### 2.3. Intervention: DHRM Framework

The DHRM framework mainly operationalizes CAS theory by treating the nursing workforce as a dynamic system of autonomous agents guided by simple rules and real‐time attractors. Dynamic staffing and surge redeployment rapidly match resources to demand, shortening waiting and collection times (operational efficiency). Dispatcher‐led coordination, fixed‐zone assignment, and instant messaging improve information flow and proximity, reducing preanalytical errors and hematomas (specimen quality and safety). Standardized protocols function as shared simple rules that ensure uniform technique, further protecting specimen integrity. Performance‐based incentives serve as key attractors that enhance motivation and fairness (nurse job satisfaction).

The foundation was a dynamic staffing algorithm adapted from Legrain et al.’s nurse‐scheduling optimization model [39], which minimizes the maximum workload across shifts while respecting coverage constraints. We extended this base formulation by incorporating real‐time EHR‐triggered demand forecasts and a risk‐adjusted safety margin (adding 10%–15% extra nurses above the calculated minimum during predicted surges) to prevent understaffing during unpredictable peaks. This modified algorithm generated tiered staffing strategies that automatically adjusted nurse deployment according to four predefined workload scenarios (baseline, moderate, high, and extreme surges). For instance, during extreme surges (300–600 specimens/day), the strategy mandated the deployment of 17‐18 nurses, including baseline staff, additional dynamic shift nurses, and redeployed outpatient nurses. These strategies utilized fixed shifts (08:00–15:00, 15:00–23:00, and 23:00–08:00) and dynamic shifts (11:00–19:00 and 12:00–20:00) that were activated specifically during surges. Outpatient nurses received training in ABG protocols to ensure a response time under 30 min.

Optimization of staff coordination and collection sequence was achieved through a dedicated dispatcher system. This system prioritized ABG requests via EHR and dynamically allocated nurses based on availability and proximity. Dispatchers underwent standardized training in crisis resource management and EHR navigation. A fixed‐zone assignment model replaced cross‐building rotation, assigning dedicated nurses to each inpatient building to improve familiarity and increase efficiency by reducing travel time. Proximity‐driven task dispatching assigned tasks to nurses within the same or adjacent zones, with accuracy maintained by periodic location updates. A secure hospital‐approved messaging platform streamlined task alerts and facilitated real‐time communication, including an escalation mechanism for tasks unresolved over 15 min.

A comprehensive standardized management protocol was developed through literature review and multidisciplinary consensus to ensure quality control and operational efficiency in ABG specimen collection and analysis. This protocol included standardized ABG specimen collection procedures (patient assessment, identity verification, culturally adapted health education, and standardized collection techniques such as radial artery as primary site, specific operational steps, and immediate gentle specimen mixing for 8–10 times). It also optimized ABG analysis workflow through dual‐check specimen verification, strict timeliness control (immediate testing within 15 min), management of nonconforming specimens (rejection criteria and corrective actions), and automated data management (HIS upload within 20 min and critical value SMS alerts).

Finally, a dynamic postperformance distribution system was established to incentivize nurses while safeguarding fairness and voluntariness. The system rested on three transparent, objectively measured indicators: working hours (manually verified), workload (quantified blood‐collection instances with bonus points for complex cases), and work quality (incidence of nursing risk events). All indicators were openly displayed on a shared dashboard each month. Compensation comprised base salary, performance pay directly tied to collection volume (“more work, more reward”), night‐shift allowance, and targeted bonuses for excellence in ABG specimen quality. Penalties for repeated risk events were strictly proportional, capped at 5% of monthly performance pay, and applied only after documented counselling and retraining. Fairness and noncoercion were ensured through (1) full transparency of scoring criteria and individual results, (2) a formal peer‐review and appeal process overseen by the nursing department head, (3) guaranteed minimum total income regardless of penalties, and (4) explicit opt‐out consent at recruitment (no nurse declined). These safeguards prevented punitive pressure and maintained the incentive system as a genuine motivator rather than a coercive tool.

### 2.4. Outcome Measures

Outcome measures in this study were categorized into three main areas: operational efficiency, specimen quality and patient safety, and nurse job satisfaction.

Operational efficiency included average patient waiting time (minutes), specimen collection time (minutes), and the incidence of delayed testing (%).

Specimen quality and patient safety comprised the first‐attempt venipuncture success rate, the specimen compliance rate, and the hematoma incidence. The first‐attempt venipuncture success rate was defined as successful blood collection of 0.5–1.5 mL on initial needle insertion or after minor repositioning. The specimen compliance rate represented specimens meeting all criteria, including the absence of air bubbles, no coagulation, and volume greater than 1 mL. Hematoma incidence was measured as the frequency of postprocedure hematoma development.

Nurse job satisfaction was assessed using the Medical Staff Job Satisfaction Assessment Scale developed by Wang et al. [40], a validated instrument specifically designed for medical personnel in Chinese hospitals (Cronbach’s *α* = 0.91 overall and subscale *α* = 0.74 − 0.89). The questionnaire was administered to all 20 participating nurses both before and after DHRM implementation and evaluates five domains (hospital management, workload, interpersonal relationships, salary and benefits, and personal development) using a 5‐point Likert scale (1 = *strongly disagree* to 5 = *strongly agree*), with higher scores indicating greater satisfaction.

### 2.5. Statistical Analysis

Statistical analysis was performed using SPSS 25.0. Normality of continuous measurement data was assessed using the Shapiro–Wilk test at *α* = 0.05, and homogeneity of variance was checked with Levene’s test. For normally distributed continuous variables, data were expressed as the mean ± standard deviation and compared using independent *t*‐tests. For nonnormally distributed continuous measurement data, median (M) and interquartile range (Q) were used for expression, and comparative analyses employed Mann‐Whitney *U* tests. Categorical variables were expressed as count (percentage) and compared using chi‐square or Fisher’s exact tests. Specifically, nurse satisfaction changes before and after DHRM implementation were analyzed using paired *t*‐tests, following verification of normality and homogeneity. Statistical significance was set at *p* < 0.05. For significant findings, effect sizes (Cohen’s d for *t*‐tests and Cramer’s V for chi‐square tests) were also calculated to support the interpretation of practical and managerial importance, using standard thresholds (Cohen’s d: small ≥ 0.20, medium ≥ 0.50, and large ≥ 0.80; Cramer’s V for 1 df: small ≈ 0.10, medium ≈ 0.30, and large ≈ 0.50).

## 3. Results

DHRM implementation produced clinically meaningful improvements across all three targeted domains, illustrating how adaptive leadership and CAS principles can simultaneously optimize operations, protect patients, and energize staff.

### 3.1. Operational Efficiency

Waiting time (*p* < 0.001, large effect) and specimen collection time (*p* < 0.001, large effect) both decreased substantially, whereas delayed testing remained unchanged (*p* = 0.334). These large effects demonstrate that real‐time, algorithm‐driven resource matching effectively eliminates peak‐demand bottlenecks without relying on downstream laboratory changes (Table 1).

**TABLE 1:** Comparison of operational efficiency.

Group	*n*	Waiting time (min)	Collection time (min)	Delayed testing incidence, *n* (%)
Observation	1800	12.54 ± 5.76	5.25 ± 1.73	31 (1.72)
Control	1800	19.23 ± 6.29	6.64 ± 2.18	39 (2.17)
*t*/*χ* ^2^		33.279	21.190	0.932
*p*		< 0.001	< 0.001	0.334
Effect size		Large	Large	Large

### 3.2. Specimen Quality and Patient Safety

First‐attempt success showed no difference (*p* = 0.221), reflecting already high baseline skill. By contrast, hematoma incidence fell significantly (*p* = 0.033, medium effect), and specimen compliance improved across clotting, air bubbles, and volume criteria (all *p* < 0.05, medium effects). These medium‐sized but clinically important gains highlight the managerial power of standardized protocols and zone‐based coordination in reducing preventable preanalytical error when technical proficiency is near ceiling (Table 2).

**TABLE 2:** Comparison of specimen quality and patient safety.

Group	*n*	First‐attempt success, *n* (%)	Hematoma incidence, *n* (%)	Specimen compliance rate, *n* (%)		
				No clotting	No bubbles	Volume > 1 mL
Observation	1800	1771 (98.39)	35 (1.94)	1775 (98.61)	1786 (99.22)	1789 (99.39)
Control	1800	1761 (97.83)	55 (3.06)	1757 (97.61)	1769 (98.28)	1775 (98.61)
*χ* ^2^		1.499	4.558	4.856	6.504	5.499
*p*		0.221	0.033	0.028	0.011	0.019
Effect size		NA	Medium	Medium	Medium	Medium

### 3.3. Nurse Job Satisfaction

Large, managerially relevant gains emerged in hospital management (*p* = 0.002), workload (*p* = 0.013), salary and benefits (*p* = 0.001), and personal development (*p* = 0.005), with total satisfaction also rising markedly (*p* = 0.010, large effect). Interpersonal relationships improved directionally yet nonsignificantly (*p* = 0.073), suggesting that structural reforms and transparent incentives primarily influence perceived fairness and control rather than social dynamics in this setting (Table 3).

**TABLE 3:** Comparison of nurse job satisfaction.

Time point	*n*	Hospital management	Workload	Interpersonal relationships	Salary and benefits	Personal development	Total score
Pre‐DHRM	20	4.18 ± 0.51	4.07 ± 0.81	4.14 ± 0.45	3.84 ± 0.76	4.09 ± 0.62	20.36 ± 3.07
Post‐DHRM	20	4.62 ± 0.27	4.62 ± 0.49	4.37 ± 0.33	4.51 ± 0.29	4.57 ± 0.36	22.67 ± 2.25
*t*		3.410	2.598	1.843	3.683	2.994	2.714
*p*		0.002	0.013	0.073	0.001	0.005	0.010
Effect size		Large	Large	Large	Large	Large	Large

Taken together, the pattern of large effects on efficiency and satisfaction, coupled with medium yet safety‐critical gains in quality, reveals DHRM’s core leadership mechanism: dynamic resource alignment and clear performance‐reward linkage create reinforcing feedback loops that drive systemic operational excellence while sustaining a motivated, human‐centered workforce.

## 4. Discussion

### 4.1. Key Findings

This study clearly demonstrates that DHRM produces substantial and clinically relevant improvements in ABG collection across all targeted domains. Waiting and collection times shortened markedly (both *p* < 0.001, large effect), aligning with broader evidence that real‐time, adaptive staffing models reduce preanalytical delays and enhance hospital‐wide throughput [7, 36, 37].

Specimen quality also improved in ways that matter to patient safety. Hematoma incidence decreased significantly (*p* = 0.033, medium effect) and compliance rates for the absence of clotting, air bubbles, and adequate volume all rose (all *p* < 0.05, medium effect). These gains are particularly important because even minor preanalytical errors can alter acid–base interpretation in up to one in six ICU patients and prompt unnecessary ventilator changes [41, 42].

From a leadership perspective, the most striking result was the large increase in nurse job satisfaction (*p* = 0.010, large effect), driven primarily by improved perceptions of workload management, compensation fairness, and personal development. This pattern contrasts sharply with conventional float‐pool approaches that often increase burnout through unpredictable assignments [11]. It shows that linking transparent performance metrics to meaningful rewards can quickly rebuild morale in high‐pressure clinical environments.

Together, these results reveal DHRM as an adaptive, CAS‐based intervention that simultaneously raises operational efficiency, strengthens patient safety, and sustains an engaged workforce. Such triple‐benefit outcomes are exactly what hospital leaders seek amid ongoing staffing shortages and rising clinical demands.

### 4.2. Mechanism Analysis: Why DHRM Outperforms Traditional Models

The exceptional performance of the DHRM framework arises from leadership competencies that are deeply woven into its core design and daily operation [43]. These competencies mainly include strategic foresight, situational adaptability, and data‐driven governance.

Strategic foresight allows leaders to look ahead and prepare for changing clinical needs rather than simply respond after problems arise. Traditional staffing models rely on fixed ratios or rigid schedules that cannot adjust to sudden shifts in patient acuity, unpredictable admissions and discharges, or surges such as postsurgical volume or emergency department overflow [26, 44]. In contrast, DHRM continuously draws on real‐time data from electronic medical records, nurse‐sensitive outcomes, admission forecasts, and the current staff skill mix [26, 44–46]. This forward‐thinking approach supports proactive workload management and early resource allocation, which markedly strengthens organizational readiness and responsiveness [33, 47].

Situational adaptability is brought to life through intelligent algorithms that adjust nurse assignments and staffing levels moment by moment. The system matches specific nurse competencies, such as critical care certification or language proficiency, to the precise requirements of each patient [35]. By closing the gap between emerging needs and available resources as soon as it appears, DHRM prevents both dangerous understaffing and wasteful overstaffing. The result is far greater resource efficiency than is possible with conventional static models [48, 49].

Data‐driven governance replaces subjective judgment calls with transparent, rule‐based algorithms grounded in objective measures of patient acuity, professional qualifications, and regulatory standards [34, 50, 51]. Every staffing decision automatically produces a clear, traceable record that supports thorough compliance reviews and performance analysis. Accountability flows seamlessly from the bedside to the executive suite. Nurses see the rationale for their assignments in real time, managers receive early warnings of emerging risks, and senior leaders gain reliable evidence linking staffing patterns to patient outcomes, enabling continual policy refinement [52, 53]. Unlike traditional systems that often leave responsibility unclear after adverse events [54], DHRM creates fully integrated processes. For example, when ABG sampling delays exceed safe limits, the system instantly reallocates qualified nurses, notifies managers with detailed reports, and flags the relevant records for review. This moves the entire organization from reactive incident investigation to proactive, transparent control of care processes.

Finally, DHRM creates a self‐reinforcing cycle by aligning economic incentives with human resource goals. Performance‐based compensation rewards both efficiency and clinical accuracy, motivating staff while protecting care quality. As a result, productivity improves alongside staff satisfaction, resolving the persistent conflict between operational pressure and workforce well‐being that plagues traditional models. Leaders thus cultivate a culture of excellence, accountability, and sustained high performance.

## 5. Limitations and Future Directions

This study has several limitations. It was conducted in a single large tertiary hospital with a short‐term pre–post design (December 2022 versus March 2023) and a homogenous cohort of 20 young female nurses (aged 22–26 years). These factors restrict generalizability to institutions with greater workforce diversity in age, gender, experience, or cultural background, as well as to smaller or less‐digitized hospitals. The strong hierarchical culture, high baseline standardization, and a junior nursing workforce highly responsive to performance incentives in our setting may have amplified the observed improvements in efficiency, specimen quality, and job satisfaction. In more heterogeneous or less supportive environments, the effects could be smaller and require substantial adaptation. Additionally, long‐term sustainability remains uncertain, with risks of algorithm decay, incentive fatigue, and dependence on mature EHR infrastructure and organizational adaptability.

Future research should adopt multicenter, longitudinal designs with diverse populations to confirm external validity and monitor long‐term performance. Special attention is needed to how leadership adaptability and organizational learning can prevent these risks: leaders can avoid algorithm decay through regular audits and frontline‐driven recalibration, while incentive fatigue can be countered by rotating reward types (monetary, recognition, and development), refreshing targets every 6–12 months, and sustaining open dialogue on fairness. Such continuous learning practices will be essential to maintain DHRM benefits over years rather than months. Additional priorities include quantifying the role of EHR maturity and institutional culture, integrating artificial intelligence for demand forecasting, and extending DHRM to other time‐critical procedures with safeguards for equity and resource‐constrained settings.

## 6. Conclusion

DHRM significantly improved ABG collection across all three domains: operational efficiency (large effect on waiting and collection times), specimen quality and safety (medium but clinically meaningful effects), and nurse job satisfaction (large effect, especially in workload control and compensation fairness). Far more than an operational tool, DHRM serves as a transformative leadership model that equips managers with strategic foresight to anticipate demand, situational adaptability to respond in real time, and transparent data‐driven governance to motivate staff through fair, performance‐linked rewards. Wider adoption and refinement through multicenter, longitudinal research will be essential to realize its full potential across diverse settings.

## Funding

The authors have nothing to report.

## Conflicts of Interest

The authors declare no conflicts of interest.

## Data Availability

The datasets used and analyzed during the current study are available from the corresponding author upon reasonable request.
